# Cross-platform Data Analysis Reveals a Generic Gene Expression Signature for Microsatellite Instability in Colorectal Cancer

**DOI:** 10.1155/2019/6763596

**Published:** 2019-03-17

**Authors:** Anna Pačínková, Vlad Popovici

**Affiliations:** ^1^Faculty of Informatics, Masarykova Univerzita, Botanická 68a, Brno 602 00, Czech Republic; ^2^Faculty of Science, Research Centre for Toxic Compounds in the Environment, Masarykova Univerzita, Kamenice 5, Brno 625 00, Czech Republic

## Abstract

The dysfunction of the DNA mismatch repair system results in microsatellite instability (MSI). MSI plays a central role in the development of multiple human cancers. In colon cancer, despite being associated with resistance to 5-fluorouracil treatment, MSI is a favourable prognostic marker. In gastric and endometrial cancers, its prognostic value is not so well established. Nevertheless, recognising the MSI tumours may be important for predicting the therapeutic effect of immune checkpoint inhibitors. Several gene expression signatures were trained on microarray data sets to understand the regulatory mechanisms underlying microsatellite instability in colorectal cancer. A wealth of expression data already exists in the form of microarray data sets. However, the RNA-seq has become a routine for transcriptome analysis. A new MSI gene expression signature presented here is the first to be valid across two different platforms, microarrays and RNA-seq. In the case of colon cancer, its estimated performance was (*i*) AUC = 0.94, 95% CI = (0.90 – 0.97) on RNA-seq and (*ii*) AUC = 0.95, 95% CI = (0.92 – 0.97) on microarray. The 25-gene expression signature was also validated in two independent microarray colon cancer data sets. Despite being derived from colorectal cancer, the signature maintained good performance on RNA-seq and microarray gastric cancer data sets (AUC = 0.90, 95% CI = (0.85 – 0.94) and AUC = 0.83, 95% CI = (0.69 – 0.97), respectively). Furthermore, this classifier retained high concordance even when classifying RNA-seq endometrial cancers (AUC = 0.71, 95% CI = (0.62 – 0.81). These results indicate that the new signature was able to remove the platform-specific differences while preserving the underlying biological differences between MSI/MSS phenotypes in colon cancer samples.

## 1. Introduction

Microsatellite instability (MSI) refers to a genetic abnormality found in many human cancers. Microsatellites are short tandem repeats of 1-6 base pairs per unit. Spontaneous mismatches or indels in microsatellites may occur during DNA replication. Such abnormalities can be recognised and repaired by the mismatch repair (MMR) genes. Cells with defective MMR gene function exhibit an abnormal length of microsatellite repeats resulting in microsatellite instable phenotype.

Traditional approach to identify patients with MSI is using a recommended panel of five markers also known as the Bethesda Panel [1]. However, a variety of other marker panels were developed to assess MSI [2, 3]. Instability detected in* ≥ *30% tested markers is designated as microsatellite-high (MSI-H). Instability detected in < 30% tested is termed microsatellite-low (MSI-L), and the absence of instability is termed microsatellite stability (MSS). Although microsatellite instable (MSI) phenotype has been reported in diverse human cancers (e.g., colon, gastric, and endometrial), it is the most frequently associated with colon cancer. Approximately 15% sporadic colon cancers manifest the MSI phenotype [4]. The MSI colon tumours have characteristic molecular biomarkers such as silencing of the MLH1 promoter by hypermethylation [5]. Other well-known contributors to MSI instability in colon cancer are MSH2, MSH6, MLH3, or PMS2 [6, 7].

In colon cancer, despite being associated with resistance to 5-fluorouracil treatment [8], MSI is a favourable prognosis marker [9, 10]. In gastric and endometrial cancer, its prognostic value is not so well established. Nevertheless, recognising the MSI tumours is of clear clinical importance and may be important for predicting the therapeutic effect of immune checkpoint inhibitors.

Nowadays, RNA-seq represents the technology of choice for gene expression analysis. Despite the benefits of RNA-seq, a wealth of expression data already exist in the form of microarray data sets. Moreover, microarray data sets were used in several studies to obtain gene expression signatures to understand the regulatory mechanisms underlying microsatellite instability in colorectal cancer [11–15]. Therefore, having a MSI gene expression signature able to remove the platform-specific differences while preserving the underlying biological differences between MSI/MSS phenotypes would be beneficial. Although MSI testing exists, it is not routinely performed on all cases. Hence a transcriptional signature may complement available clinical features with information on MSI status.

We performed a binary classification between MSI and MSS cases. Since MSS and MSI-L tumours share similar clinicopathologic features [16, 17], MSS and MSI-L populations were pooled in a single class. A new MSI gene expression signature presented here is the first to be valid across two different platforms, microarrays and RNA-seq. A simple nearest-centroid classifier was built, and its performance in terms of area under the ROC curve estimated using a 10-fold cross-validation procedure. The final classifier was validated on independent data sets representing colon, gastric, and endometrial cancers. Pathway analysis was performed for identifying enriched pathways from MSigDB.

## 2. Materials and Methods

### 2.1. Patients and Samples

The discovery set consisted of n = 552 colon cancer samples of which n = 175 were from TCGA RNA-seq [18] (discovery cohort A1) and n = 377 from Affymetrix gene expression (GEO accession number GSE39582 [19]) (discovery cohort A2).

The GSE39582 data set consists of two independent data sets. The second data set from GSE39582 (n = 87) was used as an independent validation cohort B1. Another independent validation colon cancer cohort B2 (n = 136) is from Affymetrix gene expression (GSE41258 data set from GEO database [19]).

The gastric cancer set consisted of n = 369 samples of which n = 335 were from TCGA RNA-seq [18] (cohort C1) and n = 34 from Affymetrix gene expression (GEO accession number GSE13911 [19]) (cohort C2). The endometrial cancer set consisted of n = 116 samples from TCGA RNA-seq [18] (cohort D1).

A brief summary of all data sets can be found in Table 1.

### 2.2. RNA-Seq and Microarray Data Analysis

Gene expression data were processed following standard practices in the field as follows.

In RNA-seq data sets, genes with low counts across all libraries were filtered out prior to further analysis. Read counts were normalised using Trimmed Mean of M-values normalisation procedure [25]. Differential gene expression analysis was performed using edgeR [26] generalised linear model (batch effects included in the generalised linear model). Only genes with the absolute value of log2 fold change >1 were considered as differentially expressed (adjusted* p* value < 0.05, Benjamini-Hochberg procedure [21]).

Outlier microarrays were filtered out using (*i*) 2D images for spatial bias diagnostic and (*ii*) NUSE (Normalised Unscaled Standard Errors, median (NUSE) ≤1.035) (*affyPLM *Bioconductor package [27]). Gene expression measurements were normalised using Robust Multiarray Average procedure [28] and quantile normalisation.

Two types of Affymetrix human gene expression arrays were used in this study: Human Genome U133A 2.0 (HG-U133A) and Human Genome U133 Plus 2.0 (HG-U133Plus). HG-U133A and HG-U133Plus differ from the number of probe sets presented in the chip (HG-U133A comprises more than 22,000 probe sets; HG-U133Plus comprises more than 54,000 probe sets).

### 2.3. Construction of the Gene Expression Signature for MSI Status

For the analysis, MSI-low and MSS (microsatellite stable) populations were pooled in a single class. Using four published gene expression signatures of MSI trained exclusively on microarray data sets [11–14], we identified a core MSI gene list. First, we filtered genes common to both platforms and then found differentially expressed genes (DEGs) between MSI/MSS in RNA-seq development cohort A1. A new gene expression signature was defined as the intersection of these DEGs and the core MSI gene list. To minimise redundancy of the gene expression signature, genes with the absolute value of Pearson's correlation coefficient > 0.75 either in the cohort A1 or in the cohort A2 were excluded from the final gene expression signature (if expression levels of two genes were highly correlated, only one randomly selected representative from these two genes was included in the signature). The gene expression signature was used to construct a nearest (cosine similarity) centroid-based classifier. For each sample, a score was computed as the difference between cosine distances from the sample and the centroids of the MSI and MSS classes and used for the prediction of MSI status. If the score exceeded an optimised threshold, a sample was classified as MSI. We did not construct more sophisticated classifiers to allow direct comparison with published signatures trained exclusively on microarray data sets.

### 2.4. Performance Evaluation of the Gene Expression Signature for MSI Status

The performance of the classifier was estimated using 10-fold cross-validation. As the main performance index was used in an area under the receiver operating characteristic curve (AUC) and 95% confidence intervals (CI) were computed using the DeLong's method [20] (implemented in* pROC *R-package [29]). The gene expression signature was validated on two independent colon cancer data sets: cohort B1 and cohort B2.

Only the independent microarray data sets were used for validation due to the lack of an independent publicly available colon cancer RNA-seq data set (with present MSI status).

Besides the validation on an independent colon cancer samples, we evaluated the performance of the gene expression signature on gastric and endometrial cancer samples (cohorts C1, C2, and D1).

### 2.5. Comparison with Published Signatures Trained Exclusively on Microarray Data Sets

The gene expression signature performance was also compared with published MSI gene expression signatures trained exclusively on microarray data sets.

Giacomini et al. [11] developed a 7-gene expression signature using a custom microarray. The signature was trained on colon cancer cell lines and included one probe for noncoding RNA.

Kruhøffer et al. [12] constructed a 9-gene expression signature capable of separating the MSI and MSS samples using both sporadic and hereditary nonpolyposis tumours. The Human Genome U133A 2.0 (Affymetrix) was used to measure the level of gene expression.

Lanza et al. [13] identified a signature consisting of 27 differentially expressed genes including eight miRNAs (19-gene expression signature used in comparison with a new gene expression signature; miRNAs were excluded from the analysis). Hybridisation was performed to the human 18.5k Expression Bioarray.

Tian et al. [14] developed a 64-gene expression signature for the detection of MSI phenotypes using Agilent 44K oligonucleotide array. The signature included probes without mapping to a known gene or multiple mapping probes.

The classification of samples was carried out in the same way as before (genes of the new signature were replaced by the genes from previously mentioned published gene expression signatures). DeLong's test [20] was used to compare the AUCs of the gene expression signature and published MSI gene signatures trained exclusively on microarray data sets. The correlation analysis was performed in RNA-seq development cohort A1 to detect potential multicollinearity among the genes from signatures trained exclusively on microarray data sets. The correlation was measured as the absolute value of Pearson's correlation coefficient.

### 2.6. Functional Interpretation and Pathway Enrichment Analysis of the Gene Expression Signature

A functional and biological interpretation of the 25-gene expression signature was obtained from the Database for Annotation, Visualization and Integrated Discovery (DAVID) version 6.8 [30].

To identify pathways enriched in the gene expression signature, pathway enrichment analysis was performed against MSigDB gene collections [31] using* pathEnrich *R function [32] (adjusted* p* value < 0.05, Benjamini- Hochberg procedure [21]).

### 2.7. Statistical and Survival Analysis

All statistical analyses and survival analysis were performed in R (version 3.3.1; [33]).

The prognostic value of the gene expression signature was assessed by fitting the Cox regression model in stage II and stage III cohort A1/A2 subpopulation (adjusted* p* value < 0.05, Benjamini-Hochberg procedure [21]).

## 3. Results

### 3.1. Construction and Performance Evaluation of the Gene Expression Signature for MSI Status

**Figure 1:**
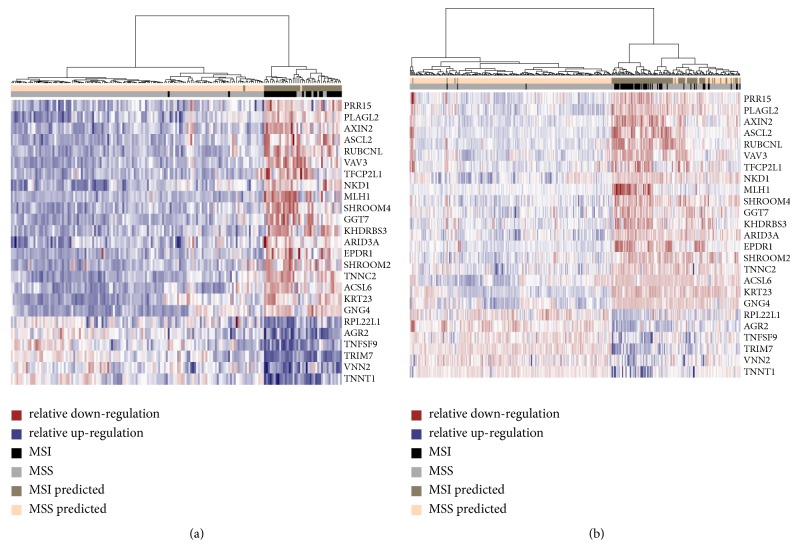
*The 25-gene expression signature profile.* (a) RNA-seq development cohort A1 (n = 175), (b) microarray development cohort A2 (n = 377). MSI microsatellite instability; MSS microsatellite stability.

We identified a new 25-gene expression signature (see Methods) (Table 2; Figure 1). In 10-fold cross-validation, the classifier performance was AUC = 0.94, 95% CI = (0.90 – 0.97) on RNA-seq cohort A1 and an AUC = 0.95, 95% CI = (0.92 – 0.97) on microarray cohort A2 (Table 3). The 25-gene expression signature was also validated in two independent microarray data sets: cohort B1 with an AUC = 0.92, 95% CI = (0.81 – 1.00) and cohort B2 with an AUC = 0.80, 95% CI = (0.70 – 0.90). Only 17 genes from the gene expression signature were used in cohort B2 (probes for eight genes were not available). We used validation cohort B2 on purpose of showing that the classifier works well also with older versions of Affymetrix microarrays. Microsatellite instable phenotype is observed in many cancers. Therefore a valid question was whether the signature could identify MSI cases also in gastric and endometrial cancer samples. The 25-gene expression signature yields good performance in gastric cancer patients both on RNA-seq data set and microarray platforms (AUC = 0.90, 95% CI = (0.85 – 0.94) and AUC = 0.83, 95% CI = (0.69 – 0.97), respectively). Furthermore, this classifier retained high concordance even when classifying RNA-seq endometrial cancer samples (AUC = 0.71, 95% CI = (0.62 – 0.81)(Table 3).

### 3.2. Comparison with Published Signatures Trained Exclusively on Microarray Data Sets

The performance of the 25-gene expression signature was compared with published signatures trained exclusively on microarray data sets (Table 3, Figure S1). The 25-gene expression signature yields better performance in comparison with Giacomini et al. [11] signature on most cohorts. On RNA-seq cohort C1, the 25-gene expression signature yields better performance in comparison with Giacomini et al. [11], Kruhøffer et al. [12], andLanza et al. [13] signatures. In case of microarray development cohort A2, the AUCs of Kruhøffer et al. [12] and Tian et al. [14] signatures were significantly better in comparison with AUC of the 25-gene expression signature.

On the contrary, the AUCs of Giacomini et al. [11] and Lanza et al. [13] signatures were significantly worse in comparison with AUC of the 25-gene expression signature on the same cohort.

**Figure 2:**
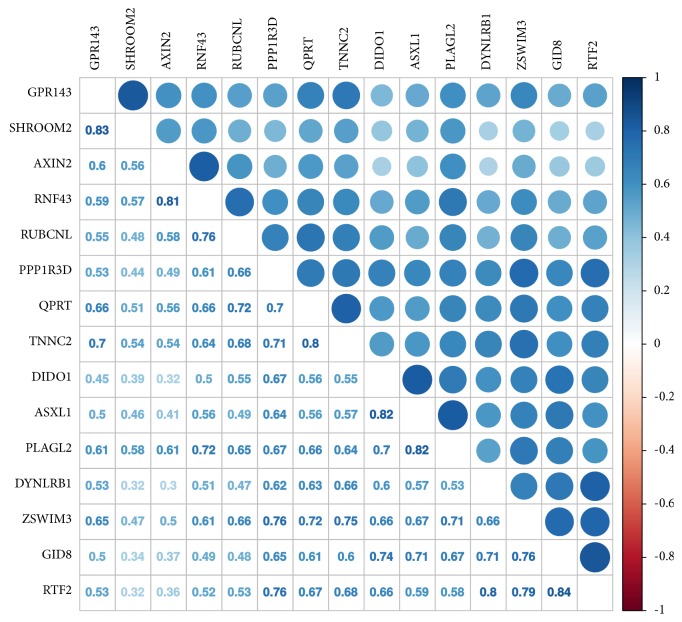
*Correlation plot of genes from the Tian et al. [14] signature with highly correlated expression levels (Pearson's correlation coefficient > 0.75) in RNA-seq development cohort A1.* The color key on the right shows the value of Pearson's correlation coefficient.

In general, the accuracy of Tian et al. [14] signature was high in all cohorts including RNA-seq development cohort A1. Therefore, we performed correlation analysis to detect potential multicollinearity among the genes from the signature in the RNA-seq development cohort A1. A high correlation between expression levels indicates the strong relationship between genes and introduces a great deal of redundancy in the signature. In the RNA-seq development cohort A1, 15 genes from the Tian et al. [14] signature had the absolute value of Pearson's correlation coefficient higher than 0.75 (Figure 2). These results suggest high redundancy of this signature in RNA-seq cohort A1.

**Figure 3:**
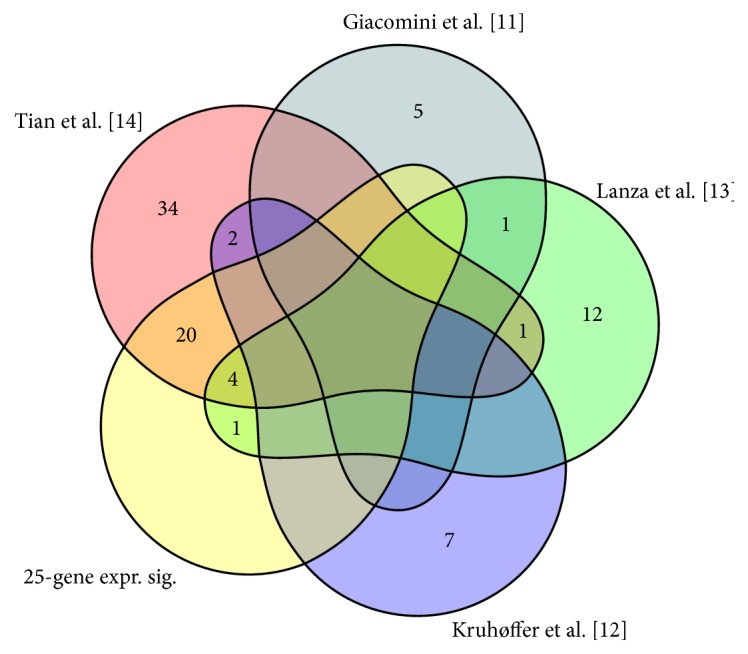
*Intersection of the 25-gene expression signature and published microarray gene expression signatures used to construct the core MSI (microsatellite instability) gene list*. 25-gene expr.sig.: the proposed 25-gene expression signature.

The intersection of the 25-gene expression signature and the published signatures is shown in Figure 3.

### 3.3. Functional Interpretation and Pathway Enrichment Analysis of the Gene Expression Signature

A functional and biological interpretation of the 25-gene expression signature was obtained from the DAVID database. Tumour-suppressor genes (MLH1 and RUBCNL), protooncogene (AGR2), and genes reported to be linked with colon cancer (EPDR1, MLH1, AXIN2) were enriched in the signature. The signature also comprised multiple genes with related oncogenic signaling pathways such as EGFR (VAV3), AKT (TNFSF9 and GNG4), or WNT (AXIN2, NKD1) signaling pathway. Genes GNG4 and VAV3 are involved in the chemokine signaling pathway that activates downstream signaling pathways such as MAPK. The 25-gene expression signature encompasses genes associated with cell differentiation, growth, adhesion, and migration.

We also carried out pathway enrichment analysis against MSigDB gene collections [31]. Three gene sets from MSigDB were significantly enriched in the new 25-gene expression signature (Table 4). The pathway enrichment analysis results support the 25-gene expression signature association with colon cancer MSI phenotype. VAV3, ACSL6, GNG4, and KRT23 were significantly enriched in gene set defined as “downregulated genes discriminating between MSI and MSS colon cancers” [22]. Results of Koinuma et al. [23] study indicate that epigenetic silencing of AXIN2 is specifically associated with carcinogenesis in MSI colorectal tumours. This is in concordance with our results.

### 3.4. Proposed Gene Expression Signature and Prognosis

We assessed the prognostic value of each gene from the proposed 25-gene expression signature by fitting the Cox regression model to identify potential drivers of the prognostic effect. Two endpoints were tested in stage II and III cohort A2 subpopulation: relapse-free survival (RFS, n = 301) and overall survival (OS, n = 304). Because of the limitation of TCGA data set, only OS endpoint was tested in stage II and III cohort A1 subpopulation (n = 115).

It is well known that patients with MSI have a more favourable prognosis compared with those with MSS. The prognostic value of the proposed 25-gene expression signature for MSI colon cancers was not statistically significant. This suggests rather than being a prognostic gene set the new 25-gene expression signature captures the underlying biological differences between MSI/MSS phenotypes.

## 4. Discussion

Carcinogenesis is a multistep process, during which genetic and epigenetic alterations determine the malignant transformation of the cell. The molecular profile of a tumour is a key determinant of clinical outcome. Therefore, the precise MSI status detection is needed for guiding the treatment strategies. Having a single MSI gene expression signature that can be used without regard to platform allows researchers to take advantage of all available microarray/RNA-seq data sets.

The main objective of this study was to identify a gene expression signature for MSI predictions in colon cancer that could be applied to both microarrays and RNA-seq data sets. We developed a new 25-gene expression signature that yields high accuracy in MSI phenotype prediction in colon cancer. Interestingly, the signature yields good performance also in gastric and endometrial cancers. From a biologic perspective, this supports the idea that MSI gene expression pattern is comparable across various cancers pointing towards similar regulatory pathways.

The 25-gene expression signature performance was also compared with published MSI gene expression signatures trained exclusively on microarray data sets. The proposed 25-gene expression signature yields better performance in comparison with Giacomini et al.'s [11] signature on most cohorts. Even if Lanza et al.'s [13] signature originally consisted of both mRNAs and miRNAs, we showed that using only mRNAs from the signature can be used to distinguish MSI/MSS colon cancer phenotypes. The accuracy of Tian et al.'s [14] signature was high in all cohorts including RNA-seq development cohort A1. However, the correlation analysis revealed high redundancy of this signature in RNA-seq cohort. Therefore, we propose the new 25-gene expression signature as a core cross-platform pattern that may form the basis for a MSI phenotype classifier across multiple cancers.

The functional annotation and the pathway enrichment analysis of the 25 genes from the new gene expression signature support the association with colon cancer MSI phenotype.

Two tumour-suppressor genes and one protooncogene were enriched in the signature. AXIN2 gene is associated with the WNT signaling pathway, and it is a direct repressor of the MYC protooncogene [34]. AXIN2 was silenced in MSI subgroup, possibly as a result of methylation of its promoter region frequently observed in MSI colon cancer patients. Interestingly, AXIN2 was also identified as one of the 36 genes that contribute to the distinction between MSI-L and MSI-H samples [35]. RPL22L1 gene was previously identified as MSI specific in gastric cancer [36] and identified as colon cancer CIMP-H subtype (characterised as enrichment for MSI, right side and mucinous histology) specific gene [37].

It should also be mentioned that MLH1 gene was previously identified as part of a gene list able to differentiate deficient/nondeficient mismatch repair phenotype in colorectal cancer samples [15].

In the microarray development cohort A2, MSI colon cancer samples with downregulated MLH1 gene expression form a compact cluster. On the contrary, MSI colon cancer samples without silencing of the MLH1 gene expression are clustered together with some MSS colon cancer samples (see dendrograms in Figure 1). Most of these MSS samples were misclassified as MSI by the proposed 25-gene expression signature. A similar pattern was observed in the RNA-seq development cohort A1. Even if these samples were predicted to be microsatellite stable, we might hypothesize they have disrupted the DNA mismatch repair system in a similar way to MSI samples without silencing of the MLH1 gene expression.

## 5. Conclusion

We present a new 25-gene expression signature able to identify MSI cases in colon cancer with consistently strong performance across microarray and RNA-seq platforms. Therefore, the new MSI gene expression signature is able to remove the platform-specific differences while preserving the underlying biological differences between MSI/MSS phenotypes in colon cancer samples. The performance of the signature on the RNA-seq data set was compared with published MSI gene signatures trained exclusively on microarray data sets. The pathway enrichment analysis results support the 25-gene expression signature association with colon cancer MSI phenotype. Moreover, the new signature is able to capture common gene activation patterns in the colon, gastric, and endometrial cancers, suggesting that the development of a common expression-based cross-platform test is feasible.

## Figures and Tables

**Table 1:** *Summary of all data sets used in the analysis.* MSI microsatellite instability; MSS microsatellite stability; HG-U133A Human Genome U133A 2.0 platform; HG-U133Plus Human Genome U133 Plus 2.0 platform.

Cohort	Tissue	Platform	MSS	MSI	Source
A1 development	colon	RNA-seq	140	35	TCGA
A2 development	colon	microarray (HG-U133 Plus)	318	59	GSE39582
B1 validation	colon	microarray (HG-U133 Plus)	77	10	GSE39582
B2 validation	colon	microarray (HG-U133 A)	107	29	GSE41258
C1	gastric	RNA-seq	281	54	TCGA
C2	gastric	microarray (HG-U133 Plus)	18	16	GSE13911
D1	endometrial	RNA-seq	64	52	TCGA

**Table 2:** List of genes in the 25-gene expression signature.

Entrez gene ID	Gene symbol	Gene description
7138	TNNT1	troponin T1, slow skeletal type
8875	VNN2	vanin 2
81786	TRIM7	tripartite motif containing 7
8744	TNFSF9	tumor necrosis factor superfamily member 9
10551	AGR2	anterior gradient 2, protein disulphide isomerase family member
200916	RPL22L1	ribosomal protein L22 like 1
2786	GNG4	G protein subunit gamma 4
25984	KRT23	keratin 23
23305	ACSL6	acyl-CoA synthetase long-chain family member 6
7125	TNNC2	troponin C2, fast skeletal type
357	SHROOM2	shroom family member 2
54749	EPDR1	ependymin related 1
1820	ARID3A	AT-rich interaction domain 3A
10656	KHDRBS3	KH RNA binding domain containing, signal transduction associated 3
2686	GGT7	gamma-glutamyltransferase 7
57477	SHROOM4	shroom family member 4
4292	MLH1	mutL homolog 1
85407	NKD1	naked cuticle homolog 1
29842	TFCP2L1	transcription factor CP2 like 1
10451	VAV3	vav guanine nucleotide exchange factor 3
80183	RUBCNL	RUN and cysteine rich domain containing beclin 1 interacting protein like
430	ASCL2	achaete-scute family bHLH transcription factor 2
8313	AXIN2	axin 2
5326	PLAGL2	PLAG1 like zinc finger 2
222171	PRR15	proline rich 15

**Table 3:** *Performance of the 25-gene expression signature and the published signatures trained exclusively on microarray data sets.* As the main performance index was used the AUC and 95% CIs were computed using the DeLong's method [20]. DeLong's test [20] was used to compare the AUCs of the published signatures and the 25-gene expression signature on a given cohort (adjusted p-value < 0.05, Benjamini-Hochberg procedure [21]). *∗* significantly better performance of the signature in comparison with the 25-gene expression signature; *∗∗* significantly worse performance of the signature in comparison with the 25-gene expression signature; 25-gene expr.sig. the proposed 25-gene expression signature; AUC area under the receiver operating characteristic curve; CI confidence interval.

	Colon				Gastric		Endometrial
	A1 development	A2 development	B1 validation	B2 validation	C1	C2	D1
	RNA-seq	Microarray	Microarray	Microarray	RNA-seq	Microarray	RNA-seq
25-gene expr.sig.	0.94	0.95	0.92	0.80	0.90	0.83	0.71
	CI (0.90 – 0.97)	CI (0.92 – 0.97)	CI (0.81 – 1.00)	CI (0.70 – 0.90)	CI (0.85 – 0.94)	CI (0.69 – 0.97)	CI (0.62 – 0.81)

Giacomini et al. [11]	0.67*∗∗*	0.56*∗∗*	0.55*∗∗*	0.69	0.63*∗∗*	0.53*∗∗*	0.47*∗∗*
	CI (0.58 – 0.76)	CI (0.49 – 0.64)	CI (0.35 – 0.75)	CI (0.59 – 0.79)	CI (0.56 – 0.71)	CI (0.33 – 0.73)	CI (0.36 – 0.58)
Kruhøffer et al. [12]	0.88	0.99*∗*	0.92	0.81	0.74*∗∗*	0.85	0.62
	CI (0.82 – 0.95)	CI (0.98 – 1.00)	CI (0.75 – 1.00)	CI (0.70 – 0.92)	CI (0.67 – 0.81)	CI (0.70 – 1.00)	CI (0.52 – 0.72)
Lanza et al. [13]	0.96	0.92*∗∗*	0.90	0.78	0.82*∗∗*	0.70	0.63
	CI (0.92 – 0.99)	CI (0.89 – 0.95)	CI (0.82 – 0.99)	CI (0.70 – 0.87)	CI (0.76 – 0.87)	CI (0.52 – 0.89)	CI (0.53 – 0.73)
Tian et al. [14]	0.97*∗*	0.96*∗*	0.95	0.82	0.89	0.88	0.71
	CI (0.95 – 1.00)	CI (0.94 – 0.98)	CI (0.86 – 1.00)	CI (0.72 – 0.92)	CI (0.84 – 0.95)	CI (0.75 – 1.00)	CI (0.61 – 0.80)

**Table 4:** *Pathway enrichment analysis of the proposed 25-gene expression signature against MSigDB gene collections.* MSigDB molecular signatures database.

MsigDB gene set name	adj. *p-*value	Genes in overlap
Watanabe colon cancer MSI vs MSS down [22]	0.005	VAV3, ACSL6, GNG4, KRT23
Koinuma colon cancer MSI down [23]	0.045	AXIN2, MLH1
Sansom WNT pathway require MYC [24]	0.045	AXIN2, NKD1, ASCL2

## Data Availability

The R code is freely available at https://github.com/bioinfo-recetox/Cross_platform_MSI_signature.

## Supplementary Materials

Supplementary Materials Supplementary Figure S1: receiver operating characteristic curves of the proposed 25-gene expression signature and the published signatures trained exclusively on microarray data sets. Supplementary Figure S2: comparison of receiver operating characteristic curves of the proposed 25-gene expression signature in RNA-seq cohorts A1, C1, and D1 normalised with different normalisation methods. Supplementary Table S3: performance of the 25-gene expression signature in RNA-seq cohorts A1, C1, and D1 normalised with different normalisation methods.
