# Supplementary material for: Cross-platform Data Analysis Reveals a Generic Gene Expression Signature for Microsatellite Instability in Colorectal Cancer
Source: Biomed Res Int. 2019 Mar 17;2019:6763596. doi: 10.1155/2019/6763596 (PMC6441508; doi:10.1155/2019/6763596)
Supplement: Supplementary Materials — Supplementary Figure S1: receiver operating characteristic curves of the proposed 25-gene expression signature and the published signatures trained exclusively on microarray data sets. Supplementary Figure S2: comparison of receiver operating characteristic curves of the proposed 25-gene expression signature in RNA-seq cohorts A1, C1, and D1 normalised with different normalisation methods. Supplementary Table S3: performance of the 25-gene expression signature in RNA-seq cohorts A1, C1, and D1 normalised with different normalisation methods. [file 6763596.f1.pdf]

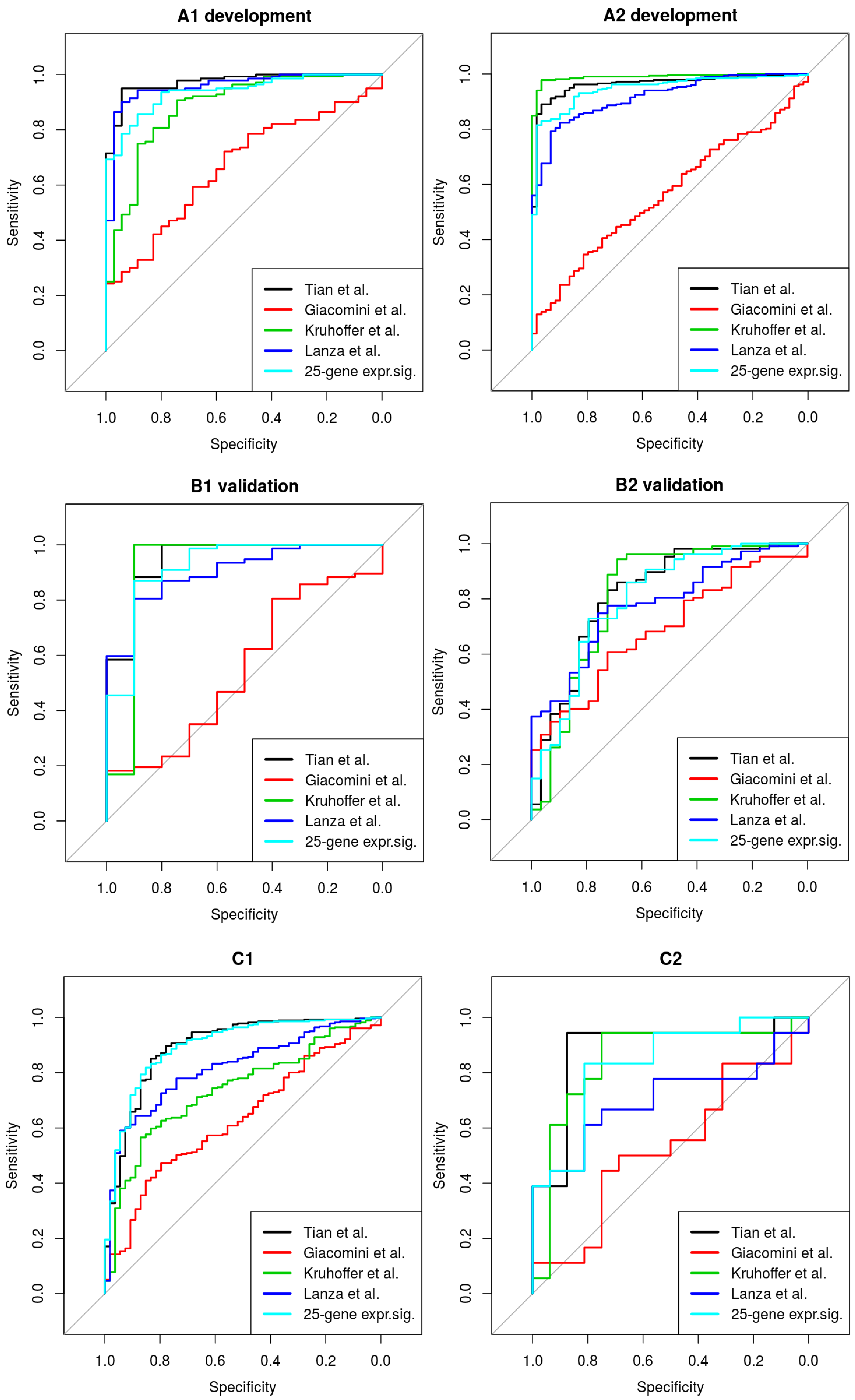

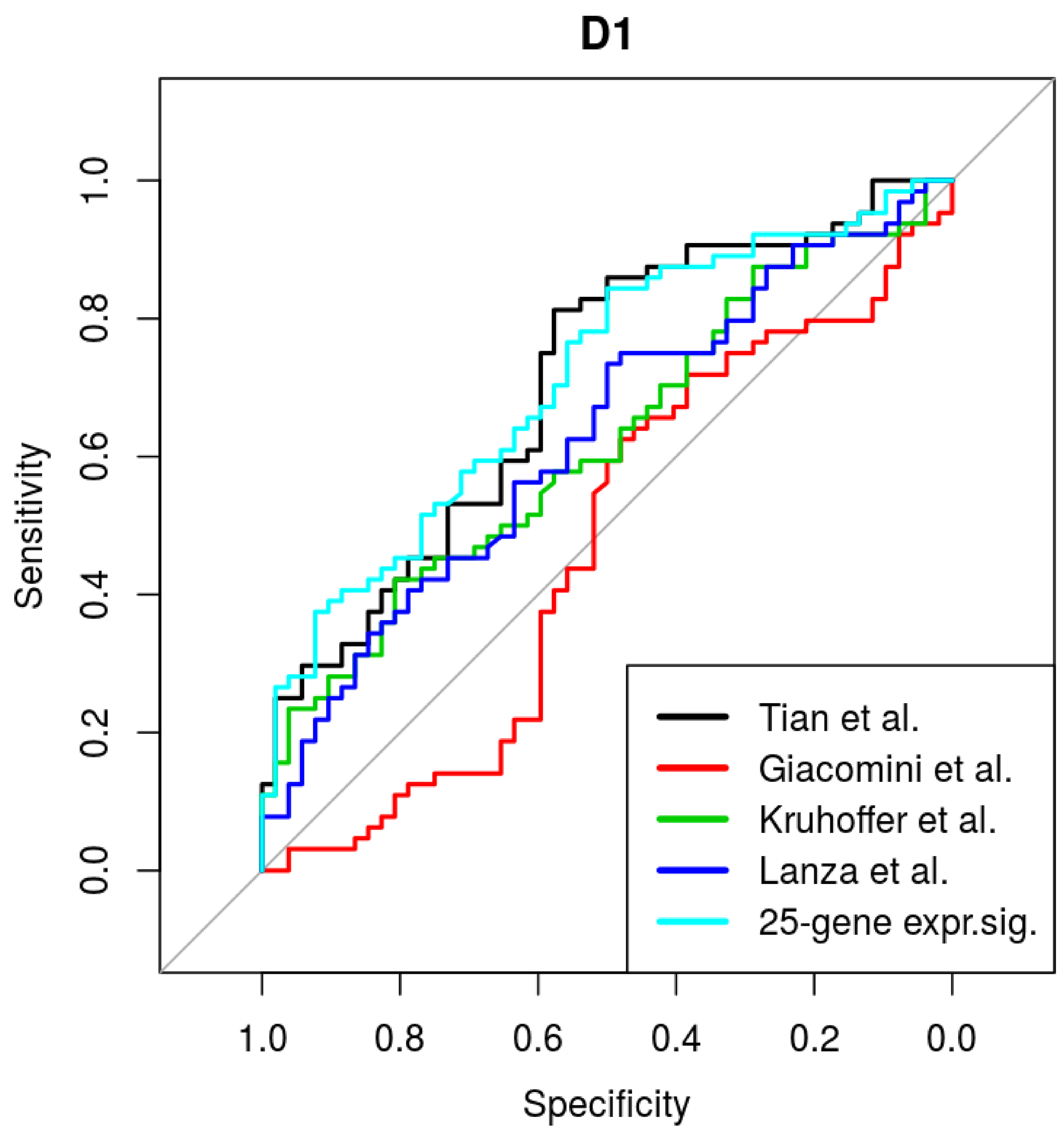

Supplementary Figure S1:

Receiver operating characteristic curves of the proposed 25-gene expression signature (25-gene expr.sig.) and the published signatures trained exclusively on microarray data sets.

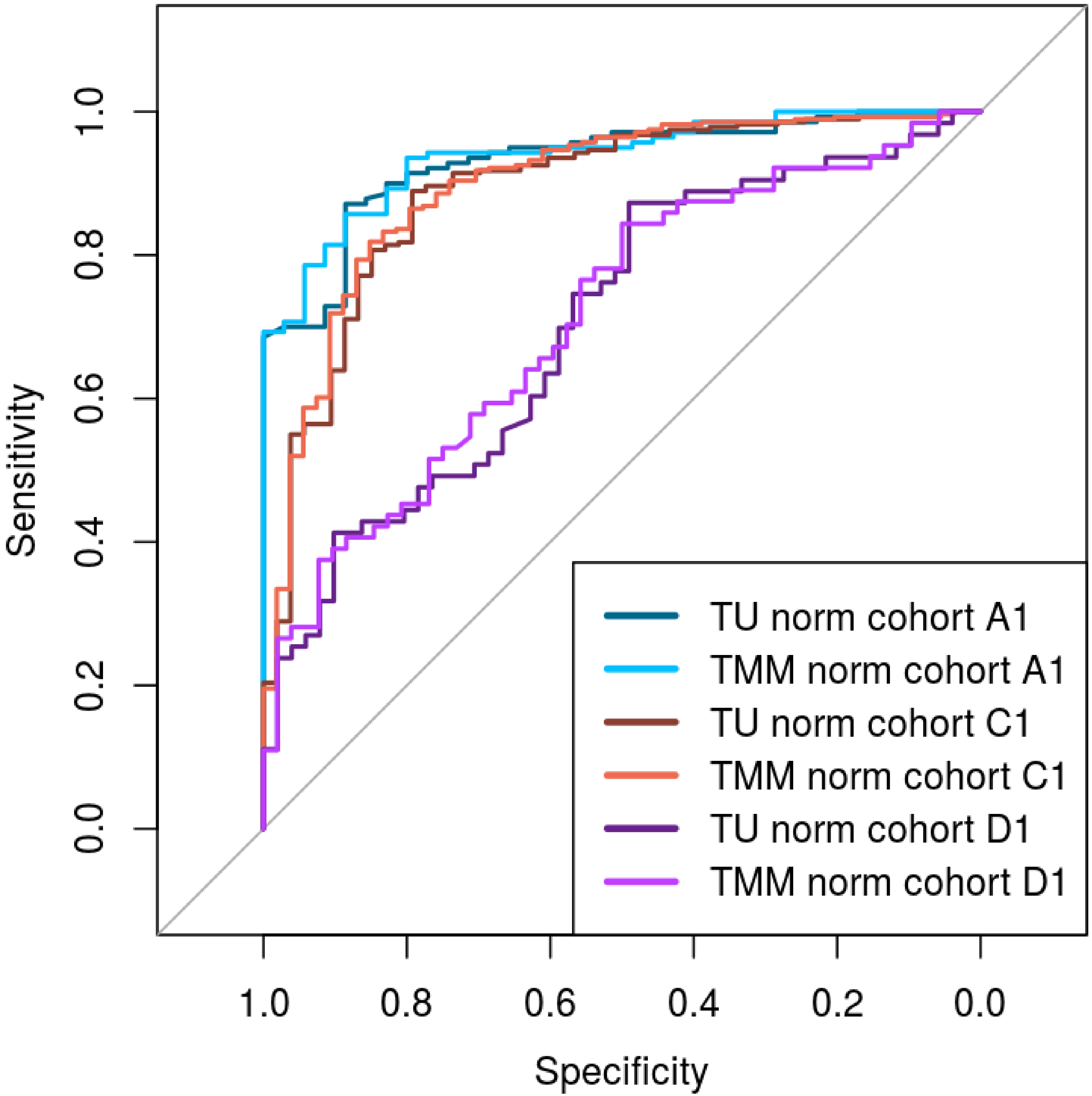

Supplementary Figure S2:

Receiver operating characteristic curves of the proposed 25-gene expression signature in RNA-seq cohorts A1, C1, and D1 normalised with different normalisation methods. TU norm (Total Ubiquitous normalisation); TMM norm (Trimmed Mean of M-values normalisation)

Glusman et al. [1] and Wu et al. [3] compared various normalisation procedures of RNA-seq data.
Based on these results, we decided to compare the performance of the proposed 25-gene expression signature on cohorts A1, C1, and D1 using TU (Total Ubiquitous) [1] and TMM (Trimmed Mean of M-values) [2] normalisation procedures. There is no significant difference in accuracy between these two normalisation methods (DeLong's test [4], adjusted p-value < 0.05).
Therefore we decided to use TMM method to normalise RNA-seq datasets to follow standard practices in the field.
NormExpression [3] R-package was used to obtain TU normalised RNA-seq data.

[1] Glusman et al. Optimal Scaling of Digital Transcriptomes. PLoS ONE. 2013
[2] Robinson & Oshlack. A scaling normalization method for differential expression analysis of RNA-seq data. Genome Biology. 2010
[3] Wu et al. NormExpression: an R package to normalize gene expression data using evaluated methods. bioRxiv preprint. 2018
[4] DeLong et al. Comparing the areas under two or more correlated receiver operating characteristic curves: a nonparametric approach. Biometrics. 1988

Supplementary Table S3: **Performance of the 25-gene expression signature in RNA-seq cohorts A1, C1, and D1 normalised with different normalisation methods.** TU (Total Ubiquitous); TMM (Trimmed Mean of M-values); AUC area under the receiver operating characteristic curve; CI confidence interval

Cohort	Normalisation	AUC	95% CI	DeLong's test *p*-value	Tissue	# genes used for classification
A1 development CV	TU	0,93	0.89 – 0.97	0,42	colon	25
	TMM	0,94	0.90 – 0.97			
C1	TU	0,89	0.84 – 0.94	0,83	gastric	25
	TMM	0,90	0.85 – 0.94			
D1	TU	0,72	0.61 – 0.80	0,90	endometrial	24
	TMM	0,71	0.62 – 0.81			
